# Nutritional and immune-related indicators-based Nomogram for predicting overall survival of surgical oral tongue squamous cell carcinoma

**DOI:** 10.1038/s41598-023-35244-y

**Published:** 2023-05-26

**Authors:** Yi-Wei Lin, Wei-Piao Kang, Chao-Qun Hong, Bin-Liang Huang, Zi-Han Qiu, Can-Tong Liu, Ling-Yu Chu, Yi-Wei Xu, Hai-Peng Guo, Fang-Cai Wu

**Affiliations:** 1 Department of Clinical Laboratory Medicine, the Cancer Hospital of Shantou University Medical College, Shantou, 515041 China; 2 Esophageal Cancer Prevention and Control Research Center, the Cancer Hospital of Shantou University Medical College, Shantou, 515041 China; 3 Department of Otolaryngology, the Second Affiliated Hospital of Shantou University Medical College, Shantou, 515041 China; 4 Department of Oncological Laboratory Research, the Cancer Hospital of Shantou University Medical College, Shantou, 515041 China; 5 The First Affiliated Hospital of Shantou University Medical College, Shantou, 515041 China; 6 Guangdong Esophageal Cancer Institute, Shantou University Medical College, Shantou, 515041 China; 7 Department of Head and Neck Surgery, the Cancer Hospital of Shantou University Medical College, Shantou, 515041 China; 8 Department of Radiation Oncology, the Cancer Hospital of Shantou University Medical College, Shantou, 515041 China

**Keywords:** Oral cancer, Tumour biomarkers

## Abstract

Oral tongue squamous cell carcinoma (OTSCC) is one of the most aggressive oral tumors. The aim of this study was to establish a nomogram to predict overall survival (OS) of TSCC patients after surgery. 169 TSCC patients who underwent surgical treatments in the Cancer Hospital of Shantou University Medical College were included. A nomogram based on Cox regression analysis results was established and internally validated using bootstrap resampling method. pTNM stage, age and total protein, immunoglobulin G, factor B and red blood cell count were identified as independent prognostic factors to create the nomogram. The Akaike Information Criterion and Bayesian Information Criterion of the nomogram were lower than those of pTNM stage, indicating a better goodness-of-fit of the nomogram for predicting OS. The bootstrap-corrected concordance index of nomogram was higher than that of pTNM stage (0.794 vs. 0.665, *p* = 0.0008). The nomogram also had a good calibration and improved overall net benefit. Based on the cutoff value obtained from the nomogram, the proposed high-risk group had poorer OS than low-risk group (*p* < 0.0001). The nomogram based on nutritional and immune-related indicators represents a promising tool for outcome prediction of surgical OTSCC.

## Introduction

Oral tongue squamous cell carcinoma (OTSCC) is one of the most frequently diagnosed tumor and the leading causes of death among head and neck cancers^1,2^. High rates of local recurrence and cervical lymph node metastasis are the most notorious clinical behaviors of OTSCC^3,4^. It has been reported that about 40–60% patients suffer from local recurrence or lymph node metastasis within 5 years^5^. Therefore, the 5-year survival rate of OTSCC patients is still unsatisfactory even with combined treatments involving surgery, radiotherapy and chemotherapy^6,7^. Owing to the diverse clinical pathological characteristics of patients, it is important to predict the outcome of OTSCC patients for the selection of more personalized treatment strategies.

At present, the TNM staging system is the gold standard for prognostication in oncology. It is based fully on the anatomical range of the disease, but it still has some limitations for survival analysis of tumor patients^8^. One of the primary disadvantages is its inability to incorporate other variables, such as genetic differences and patient characteristics including age, gender and race, to predict prognosis of cancer patients^9^. Hence, it is necessary to establish a robust prognostic model that can integrate novel prognostic factors to complement the TNM staging system to better predict the outcome of OTSCC patients. Nomogram is a reliable, user-friendly and sophisticated statistical prediction tool, with the ability to estimate individualized risk via incorporating the patient and disease characteristics^10^. Nomogram has been widely used for estimating recurrence^11,12^, specific survival^13,14^ and overall survival^15,16^ of tumor patients, and may assist clinicians in making individual treatment strategies^9^.

Studies has reported that a single blood-based indicator, such as C-reactive protein (CRP)^17^, neutrophil-to-lymphocyte ratio (NLR)^18,19^ and lymphocyte-to- monocyte ratio (LMR)^20^, served as an independent prognostic factor for survival prediction of OTSCC patients. Moreover, the prognostic nomograms which incorporated patient’s demographics and clinicopathological parameters, such as age, gender, race, tumor site and depth of tumor invasion, may also have predictive ability for the survival of OTSCC patient^3,21–28^. However, the prognostic value of detecting single marker or established nomograms based on clinicopathological parameters, seems to be insufficient for tongue cancer. In order to comprehensively improve prognostic accuracy and develop a multi-parametric prognostic model, the current study aimed to establish a nomogram to predict OTSCC patient’s outcome based on clinical characteristics and serological markers which are easy to obtain from routine admission laboratory tests, and assessed the performance of the nomogram with internal validation using a bootstrap resampling method.

## Materials and methods

### Study population and data collection

This retrospective study consisted of 169 patients with pathologically-proven OTSCC in the Cancer Hospital of Shantou University Medical College between July 2008 and February 2019. Patients were enrolled with the following criteria: (1) Tumors were confirmed as OTSCC by histopathology. (2) All patients received primary surgical resection but had not undergone chemoradiotherapy and neoadjuvant therapies. (3) Patients who suffered from any other cancers or autoimmune diseases before OTSCC diagnosis were excluded from this study. (4) All patients had complete baseline clinical information and follow-up data. This study was approved by the Hospital Ethics Committee in the Cancer Hospital of Shantou University Medical College and informed consents were obtained from all included participants. All work was complied with the principles of the Helsinki Declaration.

Clinical baseline data of each patient was collected as follows: gender, age, smoking behavior, drinking behavior, pathological TNM stage and tumor size. Clinical hematological data of the patients were collected at the time of diagnosis and before surgical treatment. The potential serum prognostic factors included creatine kinase (CK), lactic dehydrogenase (LDH), alkaline phosphatase (ALP), aspartate aminotransferase/ alanine aminotransferase (AST/ALT), total protein (TP), albumin (ALB), Albumin/Globulin (A/G), uric acid (UA), cholinesterase (CHE), immunoglobulin G (IgG), immunoglobulin A (IgA), immunoglobulin M (IgM), Complement 3 (C3), Complement 4 (C4), factor B (BF), CRP, white blood cell count (WBC), red blood cell count (RBC), hemoglobin (HGB), platelet (PLT), platelet-to-lymphocyte ratio (PLR), NLR, LMR and BMI (Body Mass Index). Tumor stage were classified according to the eighth edition of the Union for International Cancer Control/American Joint Cancer Committee (AJCC) TNM staging system^29^.

### Patients follow-up

The follow-up of patients’ survival data was acquired by retrieving medical records, email, and direct communication by mobile phone. The median follow-up time of patients was 65 months, and the minimum and maximum follow-up time was 1 month and 163 months, respectively. The overall survival (OS) was defined as the interval from the initial diagnosis to either any form of death or the last follow-up time. The last follow-up was performed in September 2022.

### Model construction and assessment

In this study, continuous variables were transformed into categorical variables and the optimal cut-off values for the continuous variables were obtained by X-tile^30^. Prognostic factors for OS were selected by Cox proportional hazards regression analysis, and those with a significant level of *p* ≤ 0.10 in univariate analysis were brought forward to multivariate Cox regression analysis. A nomogram with endpoints of 1-, 3- and 5-year OS was constructed using the prognostic factors with *p* ≤ 0.05 from multivariate Cox regression analysis. By comparing with selected prognostic factors and pTNM stage, the goodness-of-fit and discriminative ability of the nomogram were evaluated with Akaike Information Criterion (AIC) and Bayesian Information Criterion (BIC), and concordance index (C-index), respectively. Decision curve analysis was conducted to estimate the clinical utility of the nomogram, and the calibration of the nomogram was assessed with calibration curve. All internal validations were performed using bootstrapping method with 1,000 resamples.

### Statistical analyses

Statistical analyses were performed using SPSS software, version 19.0 (SPSS Inc., Chicago, IL, USA) and R (version 4.0.2) for Windows. Survival curve was plotted using Kaplan–Meier survival analysis and compared using the log-rank test with the *survminer* and *survival* in R. The nomogram, decision curve analysis curves and calibration curves were plotted by the *rms* package in R. Time-dependent C-index curves were plotted by the *pec* package in R. *p* ≤ 0.05 was considered statistically significant.

### Ethics approval and consent to participate

The authors confirm that all procedures performed in studies involving human participants were in accordance with the ethical standards of the Hospital Ethics Committee in Cancer Hospital of Shantou University Medical College and the 1964 Helsinki declaration and its later amendments or comparable ethical standards.

### Informed consent

Informed consent was obtained from all individual participants included in the study.

## Results

### Patient characteristics

The clinical characteristics of these patients were shown in Table 1. The median age of patients was 57 years (range 25–88 years), of which 93 (55%) were males and 76 (45%) were females. The numbers of patient with I-II and III-IV stage were 120 (71%) and 49 (29%), respectively. The optimal cut-off values for the continuous variables were obtained by X-tile as follows: age (69 y, range 25–88), tumor size (4.5 cm, range 0.8–6), CK (53.6 U/L, range 19.9–334), LDH (156.3 U/L, range 109.3–256.1), ALP (127 U/L, range 32.4–266.5), AST/ALT (0.96, range 0.41–1.68), TP (73.8 g/L, range 55.3–110.1), ALB (47.4 g/L, range 22.2–52.8), A/G (1.7, range 0.25–2.59), UA (261 umol/L, range 145.4–642.4), CHE (6027 U/L, range 3673–12,771), RBC (3.91 × 10^9^/L, range 2.73–6.62), HGB (130.1 g/L, range 69–169), PLT (196 × 10^9^/L, range 60–483), IgG (11.89 g/L, range 6.06–49.02), IgA (1.14 g/L, range 0.48–4.58), IgM (1.09 g/L, range 0.32–3.61), C3 (1.05 g/L, range 0.42–2.05), C4 (0.22 g/L, range 0.097–0.516), BF (0.27 g/L, range 0.2–0.92), CRP (3.78 mg/L, range 0.55–69.39), WBC (5.38 × 10^9^/L, range 3.1–12.2), LMR (2.5, range 1.36–10.2), NLR (2.96, range 0.44–7.7), PLR (177.74, range 36.98–548.86) and BMI (21.46, range 12.84–32.44).

**Table 1:** Demographics and clinical characteristics of OTSCC patients.

Characteristics	No. of patients (N = 169), n (%)	No. of events (N = 39), n (%)
Gender		
Male	93 (55)	21 (54)
Female	76 (45)	18 (46)
Age (years)		
< 69	141 (84)	29 (74)
≥ 69	28 (16)	10 (26)
Drinking		
Yes	35 (21)	27 (69)
No	134 (79)	12 (31)
Smoking		
Yes	70 (41)	18 (46)
No	99 (59)	21 (54)
Tumor size (cm)		
< 4.5	152 (90)	30 (77)
≥ 4.5	17 (10)	9 (23)
TNM stage		
I–II	120 (71)	17 (44)
III–IV	49 (29)	22 (56)
Treatment		
Surgery	121 (72)	16 (41)
Surgery followed by radiotherapy/chemotherapy	48 (28)	23 (59)
CK (U/L)		
< 53.6	22 (13)	9 (23)
≥ 53.6	147 (87)	30 (77)
LDH (U/L)		
< 156.3	76 (45)	14 (36)
≥ 156.3	93 (55)	25 (64)
ALP (U/L)		
< 127	150 (89)	33 (85)
≥ 127	19 (11)	6 (15)
AST/ALT		
< 0.96	90 (53)	18 (46)
≥ 0.96	79 (47)	21 (54)
TP (g/L)		
< 73.8	120 (71)	25 (64)
≥ 73.8	49 (29)	14 (36)
ALB (g/L)		
< 47.4	150 (89)	31 (79)
≥ 47.4	19 (11)	8 (21)
A/G		
< 1.7	108 (64)	20 (51)
≥ 1.7	61 (36)	19 (49)
UA (umol/L)		
< 261	31 (18)	11 (28)
≥ 261	138 (82)	28 (72)
CHE (U/L)		
< 6027	19 (11)	8 (21)
≥ 6027	150 (89)	31 (79)
IgG (g/L)		
< 11.89	74 (44)	21 (54)
≥ 11.89	95 (56)	18 (46)
IgA (g/L)		
< 1.14	17 (10)	7 (18)
≥ 1.14	152 (90)	32 (82)
IgM (g/L)		
< 1.09	77 (46)	20 (51)
≥ 1.09	92 (54)	19 (49)
C3 (g/L)		
< 1.05	86 (51)	16 (41)
≥ 1.05	83 (49)	23 (59)
C4 (g/L)		
< 0.22	56 (33)	11 (28)
≥ 0.22	113 (67)	28 (72)
BF (g/L)		
< 0.27	37 (22)	15 (38)
≥ 0.27	132 (78)	24 (62)
CRP (mg/L)		
< 3.78	143 (85)	30 (77)
≥ 3.78	26 (15)	9 (23)
WBC (× 10^9^/L)		
< 5.38	45 (27)	14 (36)
≥ 5.38	124 (73)	25 (64)
RBC (10^9^/L)		
< 3.91	17 (10)	8 (21)
≥ 3.91	152 (90)	31 (79)
HGB (g/L)		
< 130.1	62 (37)	17 (44)
≥ 130.1	107 (63)	22 (56)
PLT (10^9^/L)		
< 196	52 (31)	17 (44)
≥ 196	117 (69)	22 (56)
PLR		
< 177.74	146 (86)	31 (79)
≥ 177.74	23 (14)	8 (21)
NLR		
< 2.96	132 (78)	26 (67)
≥ 2.96	37(22)	13(33)
LMR		
< 2.5	22 (13)	9 (23)
≥ 2.5	147 (87)	30 (77)
BMI		
< 21.46	88 (52)	26 (67)
≥ 21.46	81 (48)	13 (33)

TNM, tumor/node/metastasis; OTSCC, oral tongue squamous cell carcinoma; CK, creatine kinase; LDH, lactic dehydrogenase; ALP, alkaline phosphatase; AST/ALT, aspartate aminotransferase/ alanine aminotransferase; TP, total protein; ALB, albumin; A/G, Albumin/Globulin; UA, uric acid; CHE, cholinesterase; IgG, immunoglobulin G; IgA, immunoglobulin A; IgM, immunoglobulin M; C3, Complement 3; C4, Complement 4; BF, B factor; CRP, C-reactive protein; WBC, white blood cell count; RBC, red blood cell count; HGB, hemoglobin; PLT, platelet; PLR, platelet-to-lymphocyte ratio; NLR, neutrophil-to-lymphocyte ratio; LMR, Lymphocyte-to-monocyte ratio and BMI, Body Mass Index.

### Construction of the nomogram

**Figure 1:**
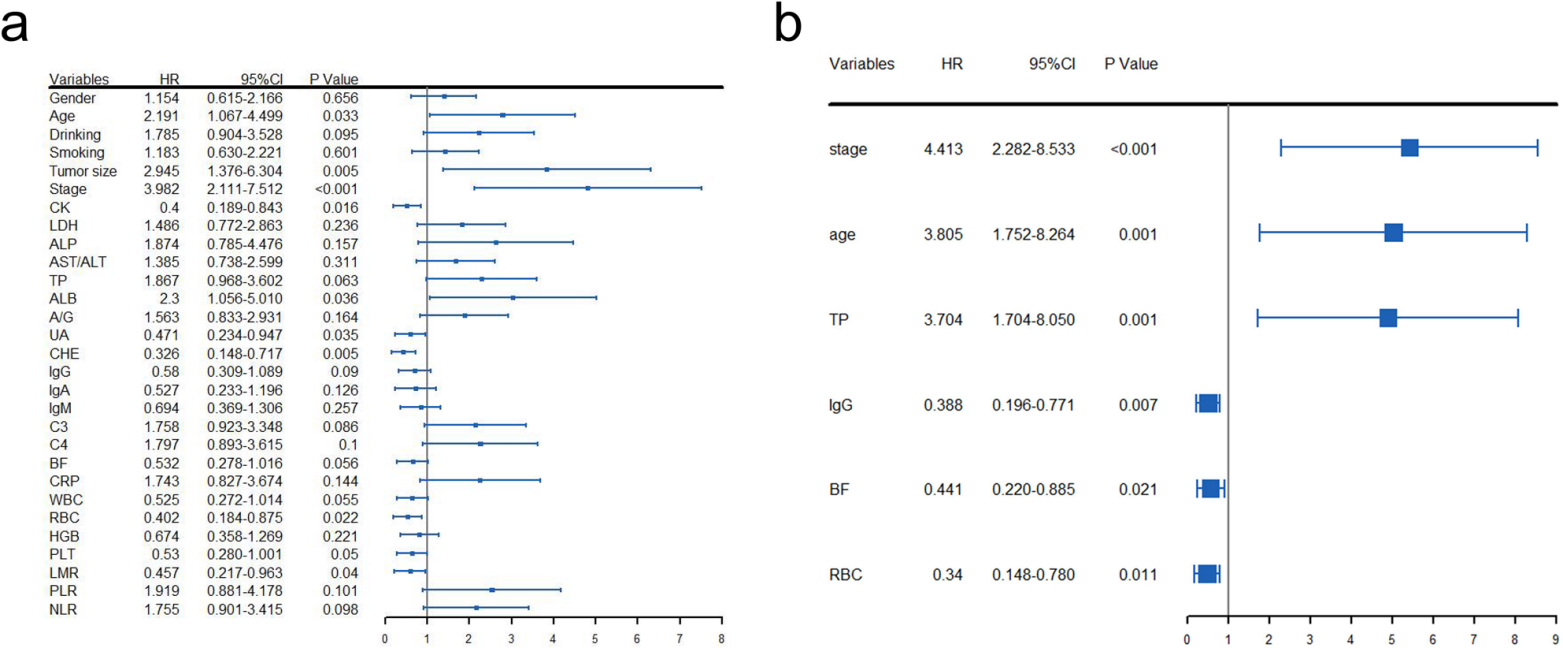
The HR and 95% CI of potential prognostic factors for OS of OTSCC patients based on the results of univariate (**a**) and multivariate (**b**) Cox regression analyses. TNM, tumor/node/metastasis; TP, total protein; IgG, immunoglobulin G; BF, factor B; RBC, red blood cell count; HR, hazard ratio; 95% CI, 95% confidence interval; OS, overall survival.

The univariate and multivariate Cox regression analyses were performed to screen out the potential prognostic markers, and to estimate their influence on OS for surgical OTSCC patients. The result of multivariate analysis showed that the following variables remained significantly independent prognostic: pTNM stage (*p* < 0.001, HR = 4.413; 95% CI: 2.282–8.533), age (*p* = 0.001, HR = 3.805; 95% CI: 1.752–8.264), TP (*p* = 0.001, HR = 3.704; 95% CI: 1.704–8.050), IgG (*p* = 0.007, HR = 0.388; 95% CI: 0.196–0.771), BF (*p* = 0.021, HR = 0.441; 95% CI: 0.220–0.885) and RBC (*p* = 0.011, HR = 0.340; 95% CI: 0.148–0.780) (Fig. 1). The detailed results of univariate and multivariate analyses are presented in Table 2.

**Table 2:** Univariate and multivariate Cox proportional hazards regression analysis for OS.

	Univariate analysis			Multivariate analysis		
	HR	95%CI	*p*	HR	95%CI	*p*
Gender (female vs. male)	1.154	0.615–2.166	0.656			
Age (≥ 69 vs. < 69; y)	2.191	1.067–4.499	0.033	3.805	1.752–8.264	0.001
Drinking (Yes vs. No)	1.785	0.904–3.528	0.095			
Smoking (Yes vs. No)	1.183	0.630–2.221	0.601			
Tumor size (≥ 4.5 vs. < 4.5; cm)	2.945	1.376–6.304	0.005			
TNM stage (III-IV vs. I-II)	3.982	2.111–7.512	0.000	4.413	2.282–8.533	0.000
CK (≥ 53.6 vs. < 53.6; U/L)	0.400	0.189–0.843	0.016			
LDH (≥ 156.3 vs. < 156.3; U/L)	1.486	0.772–2.863	0.236			
ALP (≥ 127 vs. < 127; U/L)	1.874	0.785–4.476	0.157			
AST/ALT (≥ 0.96 vs. < 0.96)	1.385	0.738–2.599	0.311			
TP (≥ 73.8 vs. < 73.8; g/L)	1.867	0.968–3.602	0.063	3.704	1.704–8.050	0.001
ALB (≥ 47.4 vs. < 47.4; g/L)	2.300	1.056–5.010	0.036			
A/G (≥ 1.7 vs. < 1.7)	1.563	0.833–2.931	0.164			
UA (≥ 261 vs. < 261; umol/L)	0.471	0.234–0.947	0.035			
CHE (≥ 6027 vs. < 6027; U/L)	0.326	0.148–0.717	0.005			
IgG (≥ 11.89 vs. < 11.89; g/L)	0.580	0.309–1.089	0.090	0.388	0.196–0.771	0.007
IgA (≥ 1.14 vs. < 1.14; g/L)	0.527	0.233–1.196	0.126			
IgM (≥ 1.09 vs. < 1.09; g/L)	0.694	0.369–1.306	0.257			
C3 (≥ 1.05 vs. < 1.05; g/L)	1.758	0.923–3.348	0.086			
C4 (≥ 0.22 vs. < 0.22; g/L)	1.797	0.893–3.615	0.100			
BF (≥ 0.27 vs. < 0.27; g/L)	0.532	0.278–1.016	0.056	0.441	0.220–0.885	0.021
CRP (≥ 3.78 vs. < 3.78; mg/L)	1.743	0.827–3.674	0.144			
WBC (≥ 5.38 vs. < 5.38; 10^9^/L)	0.525	0.272–1.014	0.055			
RBC (≥ 3.91 vs. < 3.91; 10^9^/L)	0.402	0.184–0.875	0.022	0.340	0.148–0.780	0.011
HGB (≥ 130.1 vs. < 130.1; g/L)	0.674	0.358–1.269	0.221			
PLT (≥ 196 vs. < 196; 10^9^/L)	0.530	0.280–1.001	0.050			
LMR (≥ 2.5 vs. < 2.5)	0.457	0.217–0.963	0.040			
PLR (≥ 177.74 vs. < 177.74)	1.919	0.881–4.178	0.101			
NLR (≥ 2.96 vs. < 2.96)	1.755	0.901–3.415	0.098			
BMI (≥ 21.46 vs. < 21.46)	0.504	0.259–0.980	0.044			

HR, Hazard ratio; 95% CI, 95% confidence interval; OS, overall survival; TNM, tumor/node/metastasis; CK, creatine kinase; LDH, lactic dehydrogenase; ALP, alkaline phosphatase; AST/ALT, aspartate aminotransferase/ alanine aminotransferase; TP, total protein; ALB, albumin; A/G, Albumin/Globulin; UA, uric acid; CHE, cholinesterase; IgG, immunoglobulin G; IgA, immunoglobulin A; IgM, immunoglobulin M; C3, Complement 3; C4, Complement 4; BF, B factor; CRP, C-reactive protein; WBC, white blood cell count; RBC, red blood cell count; HGB, hemoglobin; PLT, platelet; PLR, platelet-to-lymphocyte ratio; NLR, neutrophil-to-lymphocyte ratio; LMR, Lymphocyte-to-monocyte ratio and BMI, Body Mass Index.

**Figure 2:**
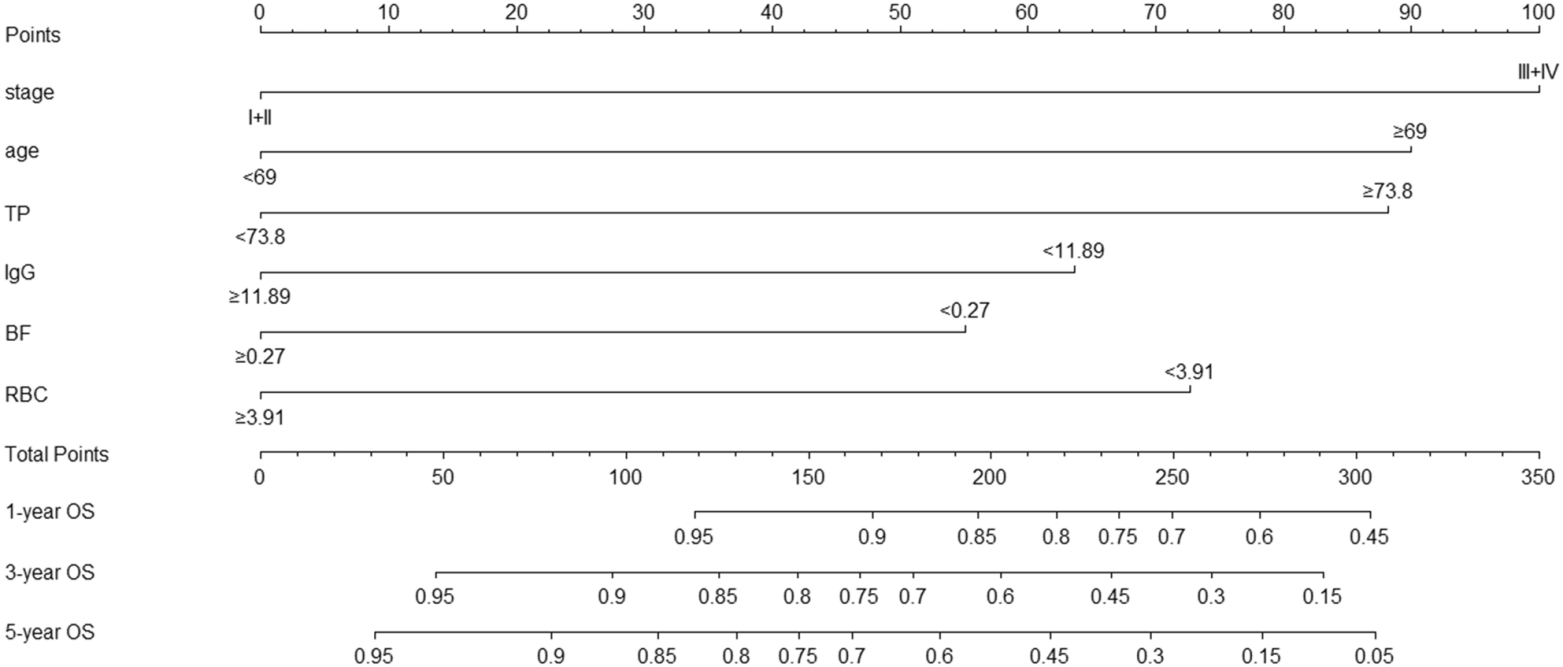
Nomogram based on TNM stage, age, TP, IgG, BF and RBC in prediction for 1-, 3- and 5-year OS of OTSCC patient. The nomogram was used by summing the points identified on the points scale for each prognostic factor. The total points projected on the bottom scales match the probability of 1-, 3-, and 5-year survival of patient. OS, overall survival; TP, total protein; IgG, immunoglobulin G; BF, factor B; RBC, red blood cell count; OTSCC, oral tongue squamous cell carcinoma; TNM, tumor/node/metastasis.

Incorporating these prognostic markers including pTNM stage, age, TP, IgG, BF and RBC, the nomogram was constructed for 1-, 3- and 5-year OS prediction (Fig. 2). From the nomogram, each factor was assigned a number of risk points, which could be obtained by drawing a vertical line directly upward from the corresponding value of the prognostic factor to an axis with “Points”. In order to determine the 1-, 3-, and 5-year OS probabilities of a specific patient, a vertical line could be drawn from the “Total Points” which was the sum of the risk points of all prognostic factors, to the axis marked “1-, 3-, and 5-year OS”. And a higher “Total Points” score would represent a worse OS for the patient.

### The goodness-of-fit and discrimination of the nomogram

**Figure 3:**
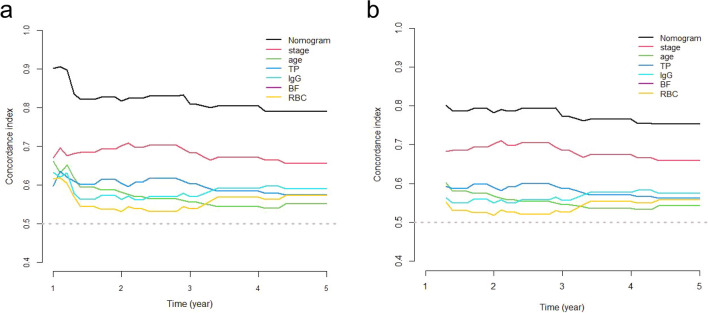
Time-dependent C-index of nomogram compared with TNM stage, age, TP, IgG, BF and RBC for OS of OTSCC patient (**a**) and internally validated with using a bootstrap resampling method (**b**). C-index, concordance index; OS, overall survival; TP, total protein; IgG, immunoglobulin G; BF, factor B; RBC, red blood cell count; OTSCC, oral tongue squamous cell carcinoma; TNM, tumor/node/metastasis.

The assessment of the goodness-of-fit and discriminative ability of the nomogram were done using the AIC and BIC, and C-index, respectively. The results were shown in Table 3. The AIC and BIC of the nomogram were much lower than those of pTNM stage (338.316 vs. 355.814; 348.298 vs. 357.478, respectively), indicating that the nomogram showed better goodness-of-fit for predicting OS. The bootstrap-corrected C-index of the nomogram was 0.794 (95% CI: 0.723–0.864), which was higher than that of pTNM stage (0.665, 95% CI: 0.589–0.741, *p* = 0.0008). Moreover, time-dependent C-index analysis also showed that the nomogram exhibited higher prognostic accuracy either for 1-, 3- and 5-year prediction OS of patient when compared with pTNM stage and any single prognostic marker (Fig. 3a). A similar result was also observed in internally validation using a bootstrap resampling method (Fig. 3b). We also conducted the ROC (Receptor Operating Curve) analysis to access the discrimination performance of the nomogram. The AUC (Area Under Curve) of the nomogram yielded 0.92, 0.83 and 0.82 for 1-, 3- and 5-year prediction OS of patients, which showed the good discrimination, better than that of the pTNM stage and any single prognostic marker (*p* < 0.001, Fig. S1). Moreover, we compared the C-indexes of DOI (Depth of Invasion) and tumor histological grading with our nomogram. The results showed that C-indexes of DOI and tumor histological grading were 0.508 and 0.568, respectively, which were lower than that of the nomogram (*p* < 0.001).

**Table 3:** The AIC, BIC and C-index of prognostic factors and nomogram for prediction OS.

	C-index (95% CI)	*p*-value	AIC	BIC
TNM	0.665 (0.589–0.741)		355.814	357.478
Age	0.561 (0.489–0.631)		369.736	371.399
TP	0.575 (0.498–0.651)		370.477	372.141
IgG	0.579 (0.500–0.657)		370.838	372.501
BF	0.569 (0.493–0.645)		370.278	371.942
RBC	0.564 (0.499–0.629)		369.339	371.003
Nomogram	0.794 (0.723–0.864)		338.316	348.298
Nomogram vs TNM		0.0008		
Nomogram vs Age		< .001		
Nomogram vs TP		< .001		
Nomogram vs IgG		< .001		
Nomogram vs BF		< .001		
Nomogram vs RBC		< .001		

C-index, concordance index; 95% CI, 95% confidence interval; OS, overall survival; AIC, Akaike Information Criterion; BIC, Bayesian Information Criterion; TNM, tumor/node/metastasis; TP, total protein; IgG, immunoglobulin G; BF, B factor; RBC, red blood cell count; *p*-values are calculated based on normal approximation using function rcorrp.cens in *Hmisc* package.

### Net benefit and predictive capacity of the nomogram

**Figure 4:**
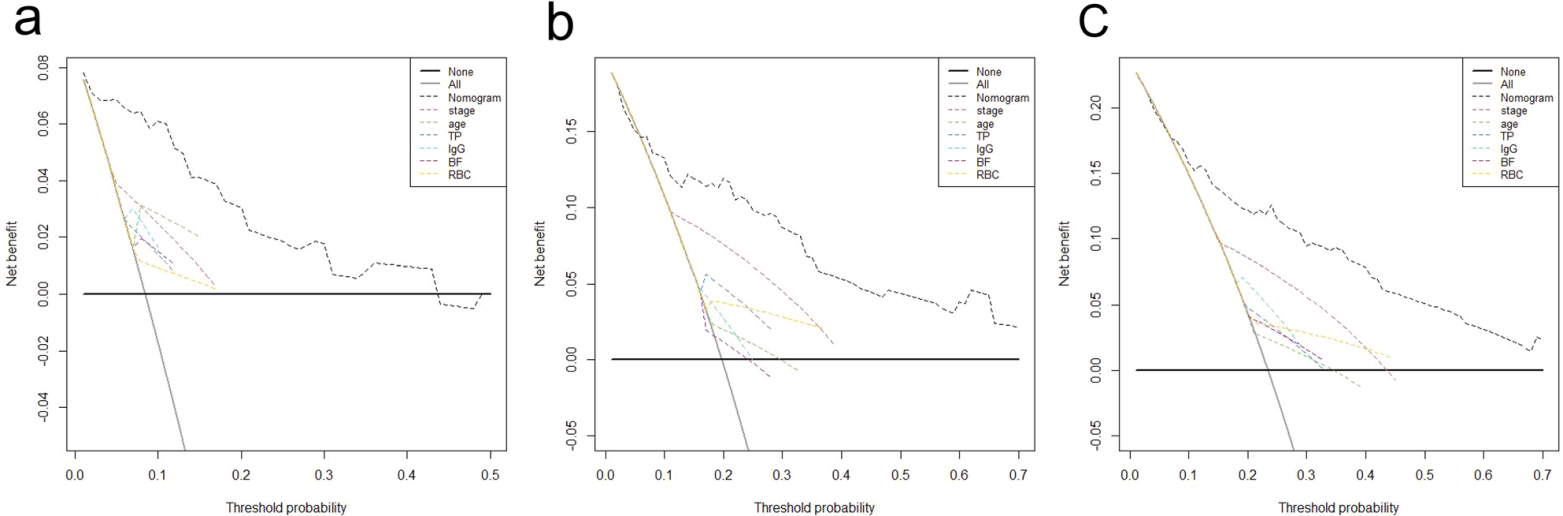
Decision curve analyses of nomogram compared with TNM stage, age, TP, IgG, BF and RBC for 1-year OS (**a**), 3-year OS (**b**), 5-year OS (**c**) of OTSCC patient. The thick grey line is the net benefit for a strategy of treating all men; the thick black line is the net benefit of treating no men. The y-axis indicates the overall net benefit, which is calculated by summing the benefits (true positive results) and subtracting the harms (false positive results). OS, overall survival; TP, total protein; IgG, immunoglobulin G; BF, factor B; RBC, red blood cell count; OTSCC, oral tongue squamous cell carcinoma; TNM, tumor/node/metastasis.

**Figure 5:**
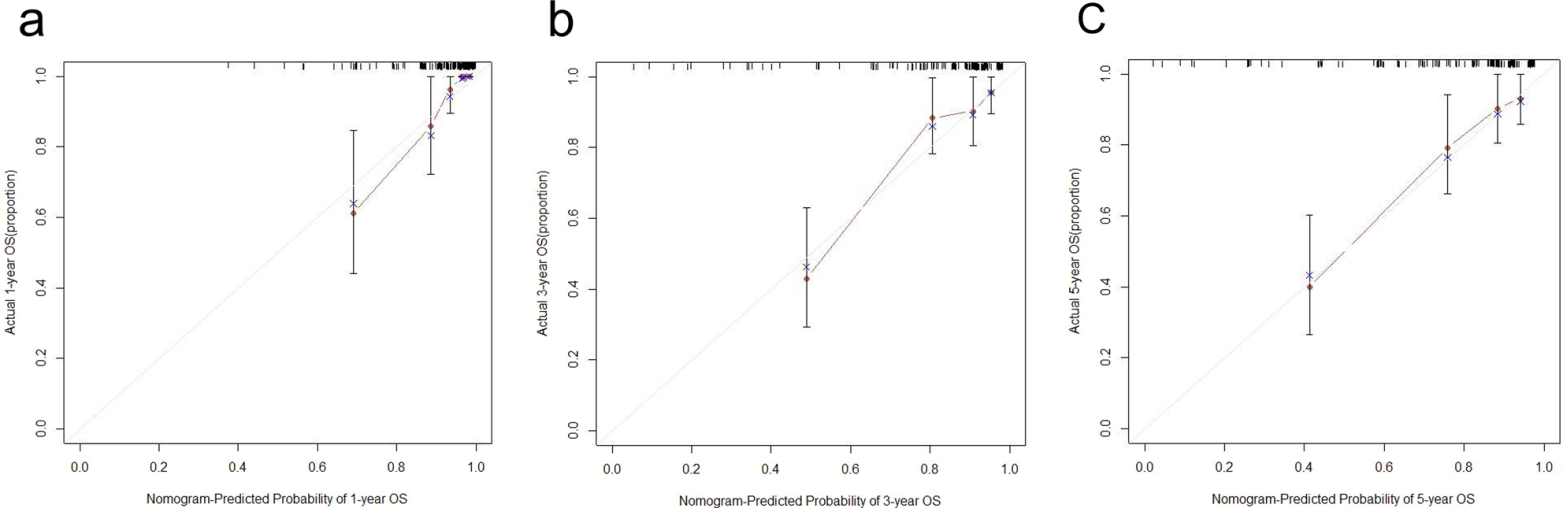
Calibration curves for 1-year OS (**a**), 3-year OS (**b**), 5-year OS (**c**) of nomogram predictions. OS, overall survival.

The decision curve analysis and calibration curve were conducted to evaluate net benefit and predictive capacity of the nomogram. As shown in Fig. 4, the decision curve analyses for 1-, 3-, and 5-year OS showed that the nomogram had improved overall net benefit compared with traditional pTNM stage across the majority of the range of reasonable threshold probabilities. In addition, calibration curve would estimate how closed the nomogram estimated risk was to the observed risk, depicted by a calibration plot. Figure 5 exhibited the good calibration of our nomogram for the 1-, 3-, and 5-year OS predictions. Taken together, these results demonstrated that our nomogram had a better performance to predict OS of OTSCC patients when compared with traditional pTNM stage system.

### Risk stratification based on the nomogram

**Figure 6:**
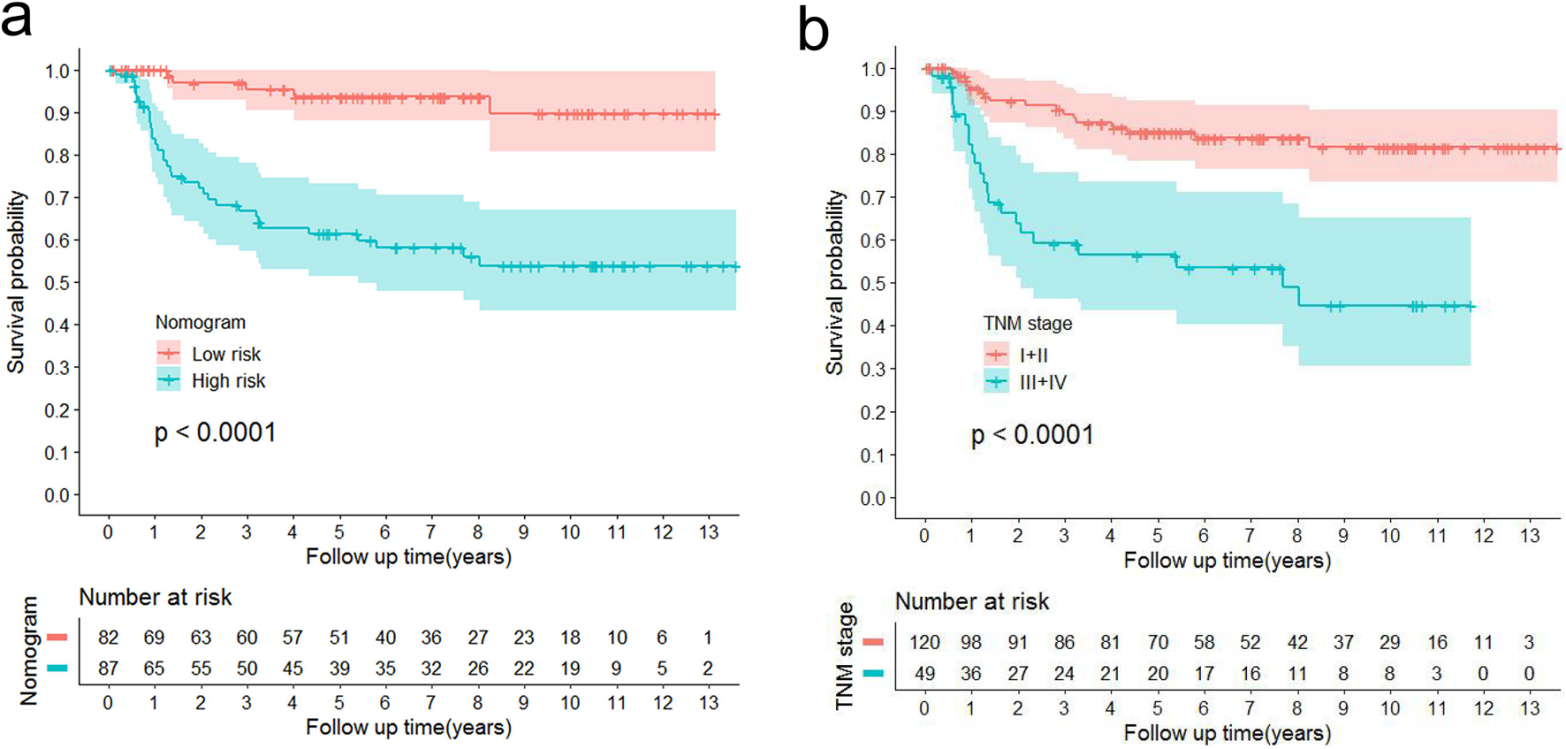
Kaplan–Meier curve for OS based on the prediction of nomogram. Low risk, Total points < 90.03 for OS; High risk, Total points ≥ 90.03 for OS. OS, overall survival.

To assess whether the OTSCC patients could be effectively separated into two proposed risk groups based on the nomogram and OS, we calculated each patient’s total risk point and used the X-tile program to obtain the optimal cutoff value. Using the cutoff value of 90.03, the OTSCC patients were subdivided into low- and high-risk groups. Then, Kaplan–Meier survival analysis was applied to assess their OS. Compared with patients in the low-risk group whose median OS was 5.93 years, patients in the high-risk group had shorter OS (median OS: 4.57 years; *p* < 0.0001; Fig. 6a), which demonstrated that the nomogram might effectively separate those patients into two risk subgroups with significant difference of OS. Moreover, we draw the survival curve of OS based on the TNM stage (Fig. 6b), and then compared with the survival curve of nomogram. From the result, the TNM stage system could also significantly predict the OS of patients as the nomogram did. However, the C-index and AUC of TNM stage were lower than that of nomogram (Fig. 3; Fig. S1), indicating that our nomogram might have higher prognostic accuracy for prediction OS of OTSCC patient.

## Discussion

OTSCC is a prevalent malignant disease characterized by high rates of metastasis and postoperative recurrence with a relatively poor prognosis^1^. At present, the prognosis and treatment of OTSCC patients are primarily determined by the AJCC TNM staging system. However, even with the same TNM stage of OTSCC, the prognosis of patients is still highly different, since it is influenced by a variety of factors^31^. Therefore, in order to find other reliable prognostic factors and help guide treatments, we established a nomogram model to predict OS of OTSCC patients with surgery by combining clinicopathological features (pTNM stage and age) and pretreatment immune- and nutrition-related indicators (TP, IgG, BF and RBC). Our nomogram showed enhanced predictive accuracy and discriminative ability when compared with the pTNM stage system. Moreover, the nomogram signature successfully separated surgical OTSCC patients into high-risk and low-risk groups with significant differences of OS.

Over the past decade, increasing researches have indicated a significant link between systemic inflammatory response and progression and prognosis of various types of tumors^32^. As a key player in the innate immune system, complement plays a dual regulatory role in the occurrence and development of tumors, affecting the outcomes of the immune response^33^. BF, as one of the complement components and a critical component of the alternative pathway amplification loop, is activated by a multitude of infectious agents including various bacteria, viruses, and fungi, in addition to neoplastic cells^34^. BF was identified with possible correlation of OTSCC patient’s outcome in this study. It has been reported that BF was associated with poor prognosis in pancreatic cancer^35–37^, and exerted a tumor-promoting role by likely initiated the PI3K-AKT or ERK1/2 signaling pathway in pancreatic cancer and cutaneous squamous cell carcinoma^35,36,38^. However, in the thyroid carcinoma, patients with higher BF expression had a longer survival compared to those with lower BF expression, mainly due to more M1 macrophages infiltrated in BF high-expression group, which implied that tumor-infiltrated macrophages exerted immune functions to exhibit anti-tumor effects^39^. To date, the correlation between OTSCC and the BF expression remains unclear. In this study, the result showed that BF was an independent prognosis factor and associated with better OS in OTSCC patients (*p* = 0.021, HR = 0.441; 95% CI: 0.220–0.885), indicating that the function of BF in OTSCC might be similar with that in the thyroid carcinoma.

Human IgG is the primary component of the human serum antibody fraction, representing about 75% of the immunoglobulins and 10–20% of the total circulating plasma proteins, and is a key component in anti-tumor humoral immune response^40,41^. Studies have demonstrated that aberrant post-translational modifications of IgG were responsible for human pathological processes including cancer^42–44^. Moreover, accumulating evidence showed that cancer-derived IgG (CIgG) are highly expressed in a variety of tumor tissues, including breast carcinoma, esophagus carcinoma, lung cancer, prostate cancer, bladder cancer, papillary thyroid cancer, colorectal cancer and pancreatic ductal adenocarcinoma. Moreover, the overexpression of CIgG was associated with poor survival outcome of tumor patients^40,45,46^. However, the characteristic of serum IgG seems totally different with that of CIgG. For example, the pre-diagnostic serum IgG level was negatively associated with the risk of melanoma or pancreatic cancer in the Swedish Apolipoprotein-related MORtality RISk cohort study^47,48^. In gastric cancer, the serum concentration of total IgG in patients was significantly lower compared with the controls, and a lower serum IgG level was closely related to poor prognosis of patients^49^. In the current study, we revealed that a higher IgG level was not only an independent prognostic factor, but was also associated with better OS in OTSCC patients (*p* = 0.007, HR = 0.388; 95% CI: 0.196–0.771). The humoral response provides a protective role against the development of tumor mediated through serum IgG, while CIgG may impede antigen-dependent cellular cytotoxicity by binding antigens and lack the capacity for complement activation^40^. The notable difference between serum IgG and CIgG manifested as restricted patterns of V(D)J recombination, which might be the reason for the different function of serum IgG and CIgG^50^.

Malnutrition is a common physical symptom in tumor patient and could be explained by a variety of mechanisms involving the tumor progression, the host response to the tumor, and anticancer therapies^51^. Increasing researches has explored the prognosis values of nutrition-related factors in cancer, such as RBC and TP ^52–54^. In endometrioid endometrial carcinoma, lower RBC level might serve as an early indicator for myometrial invasion^52^, and primary oral tumor size showed negative association with RBC count^55^. Moreover, reduced RBC count was significantly associated with poor survival outcome in colorectal cancer^56,57^ and liver cancer^58^. Similarly, our results also demonstrated that higher RBC level predicted a better OS of OTSCC patient, which was consistent with the above studies. Tumor patients frequently present with anemia, mainly due to the decreased red cell production or increased red cell destruction^59^. Those with lower RBC count are always accompanied with worse nutritional status, which may indicate the reason of prognosis value of RBC for tumor patient. In addition, TP is another nutrition-related factor, and higher TP level was associated with better nutritional status of patient^54,60^. However, in our study, the higher TP level seems to be correlated to a poor OS of OTSCC patient. This may be due to the limitation of the sample size in our study, which needs to be further explored.

Taken together, based on the results of Cox regression analysis, our nomogram consisted of six prognostic factors: pTNM stage, age, TP, IgG, BF and RBC. Our results showed that the nomogram was more accurate in predicting OS than the conventional pTNM stage system alone. The nomogram might be helpful in predicting the OTSCC patients’ outcome and treatment decisions-making. However, there are still some limitations in our study. First, it is a fact that the inflammatory marker and index included in our study could be affected by a lot of factors. In this study, the level of inflammatory marker and index were all evaluated at baseline, and thus could reduce the impact of the relevant factors. Our results showed that these markers were associated with the survival outcomes of OTSCC patients, but whether they could be applied in the clinical practice for predicting OTSCC patient outcome still needs to be confirmed repeatedly. Moreover, our nomogram not only consisted of blood test markers, but also the TNM stage. This indicated that these blood test just improved the prognostic predictive ability, but not replaced the role of TNM stage. Second, our findings were based on a retrospective design. The retrospective character of this study cannot completely exclude all potential biases. Third, patients’ data were obtained from a single cancer center, and the sample size was small. A large-scale sample from other research institutions would be required to further validate our results. Finally, the endpoint of our study was OS, and more research on the disease-free survival should also be carried out in the future. Although the above-mentioned limitations existed, our nomogram might serve as a useful tool for predicting survival outcome and helping make treatment strategies for OTSCC patients.

## Conclusions

This study established a multiparametric nomogram derived from clinicopathological features and pretreatment serological immune- and nutrition-related factors with satisfactory performance when compared with traditional pTNM stage system for individualized OS estimation. In the future, if further validation in multicenter and large-scale samples could be completed, our nomogram may be useful in clinical practice as a simple and readily available prognostic tool.

## Supplementary Information

Supplementary Legends.

Supplementary Figure S1.

## Data Availability

The data that support the findings of this study are available from the corresponding authors upon reasonable request.
