# Supplementary material for: Nutritional and immune-related indicators-based Nomogram for predicting overall survival of surgical oral tongue squamous cell carcinoma
Source: Sci Rep. 2023 May 26;13:8525. doi: 10.1038/s41598-023-35244-y (PMC10219930; doi:10.1038/s41598-023-35244-y)
Supplement: Supplementary file 1 — Supplementary Legends. [file 41598_2023_35244_MOESM1_ESM.docx]

**Fig. S1** Receptor Operating Curve analyses of nomogram compared with TNM stage, age, TP, IgG, BF and RBC for 1-year OS (**a**), 3-year OS (**b**), 5-year OS (**c**) prediction of OTSCC patient. OS, overall survival; TP, total protein; IgG, immunoglobulin G; BF, factor B; RBC, red blood cell count; OTSCC, oral tongue squamous cell carcinoma; TNM, tumor/node/metastasis
