# Supplementary figures and images for: Nutritional and immune-related indicators-based Nomogram for predicting overall survival of surgical oral tongue squamous cell carcinoma
Source: Sci Rep. 2023 May 26;13:8525. doi: 10.1038/s41598-023-35244-y (PMC10219930; doi:10.1038/s41598-023-35244-y)

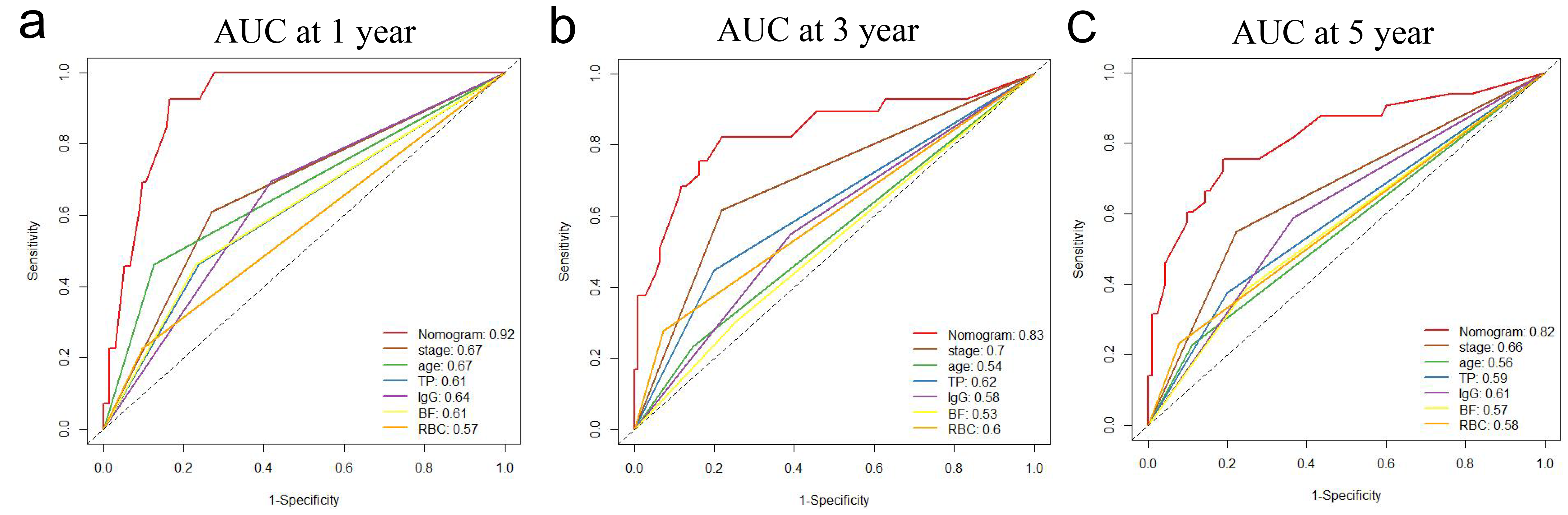

Supplement: Supplementary file 2 — Supplementary Figure S1. [file 41598_2023_35244_MOESM2_ESM.tif]
